# Evaluating the Coordination of Industrial-Economic Development Based on Anthropogenic Carbon Emissions in Henan Province, China

**DOI:** 10.3390/ijerph15091815

**Published:** 2018-08-22

**Authors:** Jianjian He, Pengyan Zhang

**Affiliations:** College of Environment and Planning, Henan University, Kaifeng 475004, China; 104753160171@vip.henu.edu.cn

**Keywords:** three major economic sectors, carbon emission, spatiotemporal difference, coordination degree index, Henan province

## Abstract

The mechanism of interaction between economic development, industrial structure and anthropogenic carbon emissions has become one of the focuses of climate change research. In this investigation, Henan Province was studied as an example, wherein the calculation model of carbon emissions in the primary, secondary and tertiary economic sectors was built using the ArcGIS 10.1 software. The spatiotemporal difference of carbon emissions between 2006 and 2015 from the three sectors was studied. The relation between economic development and environmental protection is discussed, based on the construction of a coordination degree model. Conclusions drawn from this analysis are: (1) In 2015, China’s total carbon emissions reached 10,291.93 × 10^7^ t and Henan’s carbon emissions accounted for 1.96% of China’s total carbon emissions. The total carbon emissions in Henan Province increased more than 25.00% between 2006 and 2015. (2) Carbon emissions from different economic sectors demonstrated varied patterns. The primary sector presented a gradual decreasing trend in carbon emission, while the secondary sector showed a fluctuating pattern and the tertiary sector had an inclining trend in carbon emission. (3) There are also disparities in the spatial distribution of carbon emissions from different economic sectors. The primary and tertiary sectors had higher emissions in the southeast and lower emissions in the northwest regions, while the secondary sector showed higher emissions in the northwest and lower emissions in the southeast Between cities at different prefecture levels, differences do not only lie on the quantity of carbon emissions from the three sectors of economy but also a larger variation with regards to the change in quantity of carbon emissions. (4) The coordination degree of economic development was low among different prefecture-level cities. The economic and environmental development appeared coordinated among cities at the same prefecture level; however, coordination degrees among different prefecture-level cities varies significantly.

## 1. Introduction

Since the issuance of the Third Climate Assessment Report by the Intergovernmental Panel on Climate Change (IPCC) in 2001, globe warming has become an important international political issue [1,2,3]. An increase in human industrial activity is likely to be the main cause of global climate change [4,5,6]. The global environmental problem caused by carbon emissions is concerning for sustainable development [7]. The development of a low-carbon economy to address this climate change has also become an important task for all countries in the world [8,9,10]. Carbon emissions in a country or a region are mainly decided by four factors: population, Gross Domestic Product (GDP) per capita, energy consumption per unit of GDP and energy structure [11,12,13,14]. At present, various nations have taken different measures to curb greenhouse gas emissions and developed low-carbon economy [15,16,17,18]. A low-carbon economy, which is characterized by low energy consumption, material consumption, emission and pollution, is the imperative direction for future economic development and is a strategic decision for the transformation of China’s regional economic development mode [19].

The correlation between regional economics and carbon emissions has been research extensively in various spatial extents, such as national and provisional/state levels [20,21,22,23,24,25,26,27,28,29,30,31,32,33,34,35,36,37]. However, the research results obtained by different city-level divisions are very different. Large scale (such as national and provisional/state levels) carbon emissions research highlights the overall level of regional carbon emissions but does not accurately distinguish between carbon emissions within the region; Although the division of administrative units at the county level and below is more elaborate but data acquisition is extremely difficult and the basic administrative functions undertaken by the county are not representative, which is not conducive to reflecting the basic characteristics of the region. As the political, economic and cultural unit of a country or region, cities are the high-intensity regions of human activities but carbon emission on these three economic sectors in a certain city are barely researched [19,38,39]. Therefore, the analysis of the relationship between the carbon emissions and the industrial economic development between the different cities of a province that is, to highlight the differences among the cities and shows the overall level of the province too. So, the city-level is more conducive to the development of the low carbon industry development policy.

In the past, research on coordination degree focused on industrial development or industrial development and environmental carrying capacity [40,41,42], precious few studies measure the coordination degree of industrial development from the perspective of carbon emissions. Therefore, this study compensates for the shortcomings of previous studies on the lack of coordination of carbon emissions and industrial development levels and expands the measurement field of economic development coordination. Coordinated development of regional industries means that various industries achieve interdependence, orderly operation, virtuous circle and common progress of inter-regional industries through rational division of labor and mutual cooperation. When the development between industries is uneven and the gap widens, it indicates the development of the industry. Overall coordination is reduced. The coordination degree of industrial economy from the perspective of carbon emissions is an extension of the coordination degree between economy and environment, thus, this paper defines it as a quantitative description of the degree of coupling between human carbon emissions in a certain stage of development (i.e., the carbon emissions in the process of industrial activity) and the level of regional comprehensive economic development. In China, primary, secondary and tertiary economic sectors, have been the backbone for national and regional economic development and are also the main sources of carbon emissions [35,43,44,45]. Calculating the coordination of industrial economic development from the perspective of carbon emissions is not only conducive to promoting the optimization and adjustment of regional industrial structure, promoting the upgrading of industrial energy structure but also conducive to formulating low-carbon development policies within the industry and identifying key areas for the development of low-carbon industries. Henan Province, as a major agricultural province, has the largest population and the increasing GDP. In recent years, grain production has enabled rapid social and economic development with convenient transportation. Henan has become an attractive hut for economic activities and subsequently has been faced with continuous population growth, while striving to maintain its status as a large agricultural province. In this context, its primary concern is to optimize its industrial structure and thus to improve economic and environmental benefits [33]. Carbon dioxide, as the primary greenhouse gas, is also the by-product of economic development, population growth and industrialization [14,46,47]. Curbing carbon emissions would be beneficial to maintain a favorable ecological environment; nevertheless, it would likely hinder the economic development. Thus, maintaining the equilibrium between curbing emissions and economic growth is a critical issue. 

However, it is challenging to maintain the equilibrium in China as there are distinctive disparities in economy among different regions. It requires localized strategies, which consider both local economy and environment, for curbing emissions and sustain economic growth simultaneously. China’s central government officially addressed the goals for reducing carbon emissions in 2009. The nation prospects to reduce the energy consumption per unit of GDP by 40 to 50 percent in 2020, compared with the one in 2005. Henan Province has been in a dilemma of energy conservation and higher emission demand due to economic growth. With the implementation of the to promote the rise of central China “13th Five-Year plan”, Henan Province has become one of concentrations in China’s development. In the context of growing population and maintaining large agricultural production in Henan Province, optimizing and adjusting economic structure in Henan for better economic efficiency and greater environmental benefits, have become the primary concern. Based on the differentiated calculation of carbon emission from the primary, secondary and tertiary sectors, this study discusses the spatiotemporal differences of carbon emissions in Henan Province. (1) Exploring the coordination relationship between industrial structure and carbon emissions in Henan Province, proposing regional industrial transformation mechanism and put forward low-carbon emission reduction policy suggestion; (2) Optimizing and adjusting the industrial structure and thus to improve economic and environmental benefits; (3) Promoting sustainable development in various regions of Henan Province and establishing a regional low-carbon economic system; (4) This study can also provide critical theoretical and practical reference for sustainable development and emission reduction policy formulation that are tailored for various prefecture-level cities with different local conditions in Henan Province.

## 2. Data Sources and Methodology

### 2.1. Study Area

**Figure 1:**
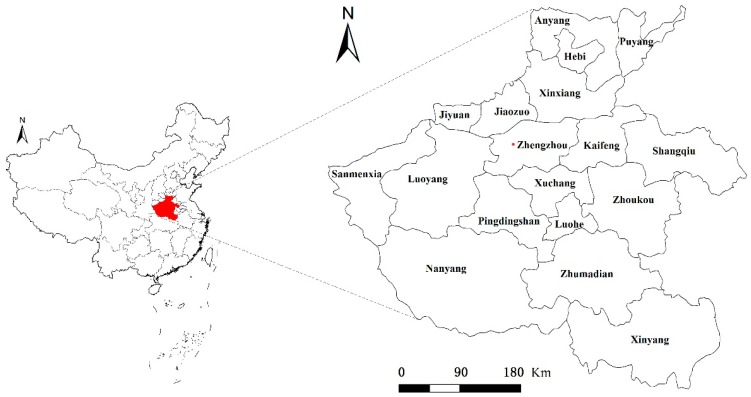
Location of the study area.

Henan Province is located in the east-central China, which is also the middle and lower reaches of the Yellow River region, with a land area of 167,000 square kilometers. According to ‘Henan Statistical Yearbook’ of 2016 (2015 data), Henan province’s GDP is 3700.21 billion yuan, of which the GDP of the primary, secondary and tertiary sectors are 420,961,791.74 and 1487.52 billion yuan. The GDP proportion of the three major economic sectors is 11.4:48.4:40.2. The GDP per capita is 39,123 yuan. The area of arable land in Henan Province is 8.13 million hectares that accounts for 48.66% of the province’s total land area (Figure 1).

Henan Province is China’s major agricultural province. However, urbanization and industrialization have intensified, especially since Henan being identified as part of the Central Plains Economic Zone. The rapid economic development has introduced great challenges and threats to the local ecological environment. A sustainable development among the economy, society and environment with good coordination is a critical issue in Henan Province.

### 2.2. Data Sources

The industrial structure of this study can be divided into the primary sector (including the crop-plantation, animal husbandry and fishery), the secondary sector (including the industrial production sectors), the tertiary sector (including the service sector). The data relate to carbon emission from the primary sector in Henan Province can be explained as three categories: (1) Agricultural capital investment, which includes fertilizers, pesticides, agricultural film, irrigation area and crop acreage. Chemical fertilizers can be further segmented into nitrogen fertilizers, phosphate fertilizers and potash fertilizers. The nitrogen fertilizer is derived by calculation of indirect carbon emissions and the direct carbon emissions during its application. (2) Crop yield, which includes rice, wheat, corn, other cereals, soybean, other beans, sweet potatoes, peanuts, rapeseed, sesame, cotton, tobacco, vegetables, mushrooms and fruits. (3) Livestock breeding and the area of fishery. Livestock breeding is the inventory of yearly animals’ intestinal fermentation and fecal carbon emissions of livestock species, including cattle, horses, donkeys, mules, pigs, goats, sheep, rabbits and poultry. The area of fishery industry is mainly the fish farming areas that were used to calculate the carbon emissions during the aquaculture process. The data source for estimation of carbon emissions from the primary sector mainly came from the 2007–2016 ‘Henan Statistical Yearbook’ and ‘China Rural Statistical Yearbook’.

Carbon emissions in the secondary and tertiary sectors were primarily estimated by the energy consumptions in the secondary and tertiary sectors. The types of energy consumption mainly include raw coal, coke, crude oil, diesel oil and fuel oil. The IPCC energy consumption carbon emission calculation coefficient was combined with the actual situation of local energy use in China and Henan Province. These were then used to estimate the carbon emissions from the secondary and the tertiary sectors in Henan Province. The energy consumption data for carbon emission estimation is mainly based on the segmented energy consumption provided by the ‘Henan Statistical Yearbook’ of 2007–2016 [48] and the basic situation of various energy uses in Henan Province are offered by the 2007–2016 ‘China Energy Statistical Yearbook’ [49].

### 2.3. Methodology

#### 2.3.1. Carbon Emissions Estimation Methods for Primary Sector

- Carbon emission in agricultural capital investment

Agricultural capital investment is the main source of carbon emissions in the agricultural sector [50,51,52,53]. The agricultural capital investment in this paper is presented as two categories: (1) the direct and indirect carbon emissions caused by chemical fertilizers (nitrogen fertilizers, phosphate fertilizers, potash fertilizers), pesticides, agricultural films and other agricultural chemicals; (2) the indirect carbon emissions resulting from agricultural irrigation and agricultural daily farming. Direct and indirect carbon emission coefficients are selected based on previous studies. [54,55,56,57,58].

(1) $$E_{ICAM}=\overset{n}{\underset{i=1}{\sum }}AM_{i}\times \alpha_{i}$$

Here, $E_{CAM}$ represents the indirect carbon emissions from agricultural capital investment and $AM_{i}$ represents the different types of agricultural capital investment and $\alpha_{i}$ is the carbon emission index for different agricultural capital investment (see Table 1).

Both indirect carbon emissions released in the process of fertilizer application and the direct carbon emissions caused by its application are taken into account [59]. The direct carbon emissions generated during the application of nitrogen fertilizers in agricultural processes are 20% greater than the indirect carbon emissions [60,61].

(2) $$E_{CN}=F_{N}\times \alpha \times \beta$$

Here, $E_{CN}$ is the direct carbon emission from nitrogen fertilizers, $F_{N}$ is the amount of nitrogen fertilizer being applied, *α* is the conversion coefficient of nitrogen to carbon and *β* is the direct carbon emission coefficient of nitrogen fertilizers. According to the existing research results [62,63], the nitrogen to carbon conversion coefficient and the direct carbon emission coefficient of nitrogen fertilizers in Henan Province are 81.27 t C/t N_2_O and 0.0057 kg N_2_O-N/kg N, respectively.

- Carbon emissions from straw combustion

Crop straw is an inevitable subsidiary product in the agricultural production process with a large production volume. The straw is mainly disposed by combustion and its combustion process is still an important carbon source from agricultural activities.

(3) $$E_{CS}=P_{i}\times S_{i}\times SC_{i}\times \theta_{i}$$

Here, $E_{CS}$ is the carbon emission from the burning of straw, $P_{i}$ is the yield of different crops, $S_{i}$ is the straw ratio of different crops (the ratio of straw production to crop yield) and $SC_{i}$ is the crop straw collection coefficient (the ratio of crop straw collection to total straw yield) and $\theta_{i}$ is the carbon emission coefficient of straw combustion (carbon emissions during combustion per unit of straw). Based on the previous research results [64,65,66], the analysis summarizes the carbon emission-related coefficients of straw combustion for various crops in Henan Province (see Table 2). The combustion of crop straw is divided into two parts: power generation combustion (3.53%) and domestic fuel (13.5%), with a total straw burning rate of straw of 17.03% in Henan Province.

- Carbon emissions from livestock and fisheries

In terms of livestock, carbon emissions from livestock farming are mainly in the form of animal intestinal fermentation CH_4_ emissions and fecal CH_4_, N_2_O emissions. According to prior research results [62,63,67,68], the CH_4_ emission coefficient of intestinal fermentation and the CH_4_ and N_2_O emission coefficients of fecal management (Table 3) were used to calculate the carbon emissions from livestock in Henan Province.

(4) $$E_{H}=A_{i}\times \left(I_{CH_{4}}+F_{CH_{4}}+F_{N_{2}O}\right)$$

Here, $E_{H}$ is the total carbon emission from livestock and $A_{i}$ represents one of the many different livestock. $I_{CH_{4}}$ is the CH_4_ coefficient of an animal’s intestinal fermentation, $F_{CH_{4}}$ is the fecal CH_4_ emission coefficient and $F_{N_{2}O}$ is the fecal N_2_O emission coefficient. Among them, there is 6.82 tons of carbon per ton of CH_4_ and 81.27 tons of carbon per ton of N_2_O.

Considering the entire life cycle of a fishery, the carbon emissions in the aquaculture process are mainly due to the indirect carbon emissions from oxygenation in fishponds and water changing in fish tanks. According to earlier studies [69], considering that the main fishery in Henan Province engages in freshwater aquaculture, the carbon emission can be calculated using the formula:

(5) $$E_{F}=F_{A}\times \left(\mu +\rho \right)$$

where, $E_{F}$ is the total carbon emission resulting from fishery in Henan Province, $F_{A}$ is the area of fish farming, *μ* and *ρ* are the carbon emission coefficients during the process of oxygenation and water changing respectively.

Therefore, the total carbon emissions *Ec* from the primary economic sector in Henan Province can be expressed as:

(6) $$E_{C}=E_{ICAM}+E_{CN}+E_{CS}+E_{H}+E_{F}$$

#### 2.3.2. Estimation of Carbon Emissions from the Secondary and the Tertiary Economic Sectors

The carbon emission of the secondary and tertiary economic sectors was accompanied by the production and consumption of energy. Therefore, the carbon emission estimation model which was based on energy consumption was used to estimate the carbon emissions generated by the secondary and tertiary economic sectors. According to the 2006 Guidelines for National Greenhouse Gas Inventories [70] prepared by the IPCC, the carbon emissions calculation method can be divided into total energy consumption, energy balance and terminal energy consumption. In order to maintain consistency with the World Bank’s global carbon emissions database, the total energy consumption was used to estimate the carbon emissions of energy consumption in Henan Province. Derived from the industrial energy consumption combined with the energy-related carbon emission indicators in Henan Province, the carbon emissions from these sectors were estimated. This assessment was done using the provisions of the economic sectors (with the secondary sector mainly consisting of industrial energy consumption and the tertiary sector incorporating transportation, warehousing, postal services, wholesale and retail, accommodation and catering), with reference to former research [71,72,73] and by implementing the IPCC recommended carbon emissions calculation guidelines [62].

(7) $$E_{T}=\overset{n}{\underset{i=1}{\sum }}EM_{i}\times LCV_{i}\times CF_{i}\times O_{i}$$

Here, $E_{T}$ denotes the carbon emissions from the secondary and tertiary economic sectors of Henan Province, *i* represents different types of energy sources, $EM_{i}$ represents the total consumption of the *i*-th energy source, $LCV_{i}$ represents the low combustion value of the *i*-class energy, $CF_{i}$ denotes the carbon emission coefficient of the *i*-th energy source and $O_{i}$ denotes the combustion oxidation rate of the *i*-th energy source (see Table 4).

#### 2.3.3. Industrial-Economic Development Coordination Degree

The study of economic-environmental coordination has a long history and such research mainly estimates the relationship between the economic and environmental development from the perspective of the economic development and ecological environment bearing capacity. This study analyzes the development coordination degree of the industry (carbon emissions from the primary, secondary and the tertiary sectors) and the economy (the GDP from the primary, secondary and the tertiary sectors) from the carbon emission point of view and puts forth a new coordination degree measurement method based on the previous research [74].

(8) $$H\left(0<H\leq 1\right)=\frac{\sum_{j=1}^{n}S_{j}C_{j}}{\sqrt{\sum_{j=1}^{n}S_{j}^{2}\sum_{j=1}^{n}C_{j}^{2}}}$$

where, H is the coordination degree coefficient between the industrial carbon emission and economic production structures, $S_{j}$ is the proportion of production value from the primary, secondary and tertiary economic sectors in different regions and $C_{j}$ is the proportion of carbon emissions from the primary, secondary and tertiary sectors in different regions, n represents the primary, second and tertiary industry. Coordination coefficient can reflect the proportional relationship between industrial structure and carbon emission structure development as a whole and the value range is (0,1). When the coordination coefficient is 0, it indicates that the coordination between industrial structure and carbon emission structure is poor; when the coordination coefficient is 1, it indicates that the two have a good proportional structure, indicating that the industrial structure is similar to the carbon emission structure, that is, the industrial structure has a similar development law to carbon emission structure.

## 3. Results and Discussions

### 3.1. Time Series Estimations of Carbon Emissions in Three Economic Sectors in Henan Province

Industrial activity is one of the main sources of carbon emissions [14,43,44], the uncertainty of carbon emission estimation is an important issue in this field, which mainly reflects the two aspects of calculation method and carbon emission factor selection. On the basis of selecting the energy consumption as the calculation method of carbon emission, the selection of carbon emission factors makes the total amount of regional carbon emissions greatly different, which is related to the regional energy nature [7,75]. Compare the IPCC with the carbon emission factors of the eight energy sources in this study, where the carbon dioxide emissions per kilogram of crude coal differ by 0.87 km (see Table 5). Taking the calculation of the total carbon consumption of energy consumption in Henan Province in 2015 as an example, the total carbon emission estimated by the IPCC carbon emission coefficient is 23.85 × 10^7^ tons higher than this study. The difference is mainly due to lower heating value, carbon content and Oxidation rate. Different choices indicate that the uncertainty of carbon emission factors will have an important impact on the total carbon emissions [7,75]. Selecting the carbon emission coefficient calculated by this study to estimate the carbon emissions of energy consumption will be able to scientifically and accurately assess the actual situation of carbon emissions from energy consumption in Henan Province and is more conducive to measuring the industrial coordination degree from the perspective of carbon emission.

**Figure 2:**
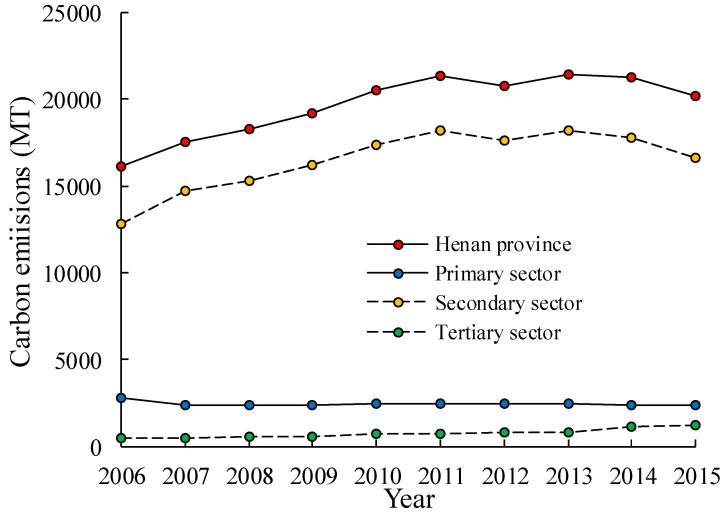
Variation of carbon emissions over time from three major economic sectors in Henan Province during 2006–2015.

Carbon emissions in three major economic sectors in Henan Province during 2006–2015 are illustrated in Figure 2. It shows that both total carbon emissions and the carbon emissions only in the secondary sector tended to increase first until around 2011 and then followed by a declining trend. The total carbon emissions increased from 161.02 million tons in 2006, to 213.40 million tons in 2011 and reduced to 202.03 million tons in 2015. The carbon emissions in the secondary sector increased from 128.49 million tons in 2006, to 181.87 million tons in 2011 and later reduced to 166.4666 million tons in 2015. Carbon emissions in the tertiary sector presented a gradual increasing trend over the study period. It increased from 4.42 million tons in 2006, to 12.26 million tons in 2015. Interestingly, carbon emission in the primary sector tended to be decreasing gradually, from 28.12 million tons in 2006, to 23.30 million tons in 2015. The percentage contribution of the primary, secondary and tertiary sectors towards the total carbon emissions in Henan Province were 11.00%, 82.00% and 3.00%, respectively. It indicates that the secondary sector, which contributes the most, is the main source of carbon emissions in Henan Province. 

**Figure 3:**
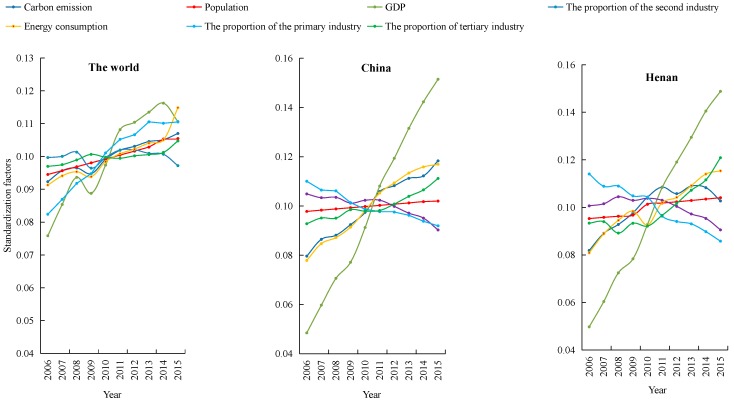
Variation of carbon emissions over time from three major economic sectors in Henan Province in comparison to China and the World during 2006–2015.

Figure 3 shows the changes in Henan Province carbon emission and its influencing factors. In 2015, China accounted for 28.48% of the world’s total carbon emissions, Henan’s carbon emissions account for 1.96% of China’s total carbon emissions. Compared with 2015, the growth rate of carbon emissions in China and Henan Province was 1.28 times and 1.08 times higher than the world average in 2006; the proportion of the tertiary economic sector in the world, China and Henan Province was 70.12%, 50.19% and 40.20% respectively, however, the proportion of the secondary economic sector in China and Henan Province was 13.85% and 21.34% higher than that of the world’s second economic sector, this indicated that the difference of industrial structure had an important impact on the growth of regional carbon emissions. The implementation of the strategy of “Central Plains Economic Region” in 2012 had made significant changes in the industrial structure of Henan province, 2012–2015, the proportion of the primary and secondary economic sector decreased by 1.10% and 5.29% respectively. The rate of decline was significantly higher than that of 2006–2011 and the change in carbon emissions was consistent with the primary and secondary economic sector.

### 3.2. Spatial Distribution Characteristics of Carbon Emissions from the Three Economic Sectors in Henan Province

#### 3.2.1. Spatial Distribution Characteristics of Carbon Emissions from the Primary Sector in Henan Province

**Figure 4:**
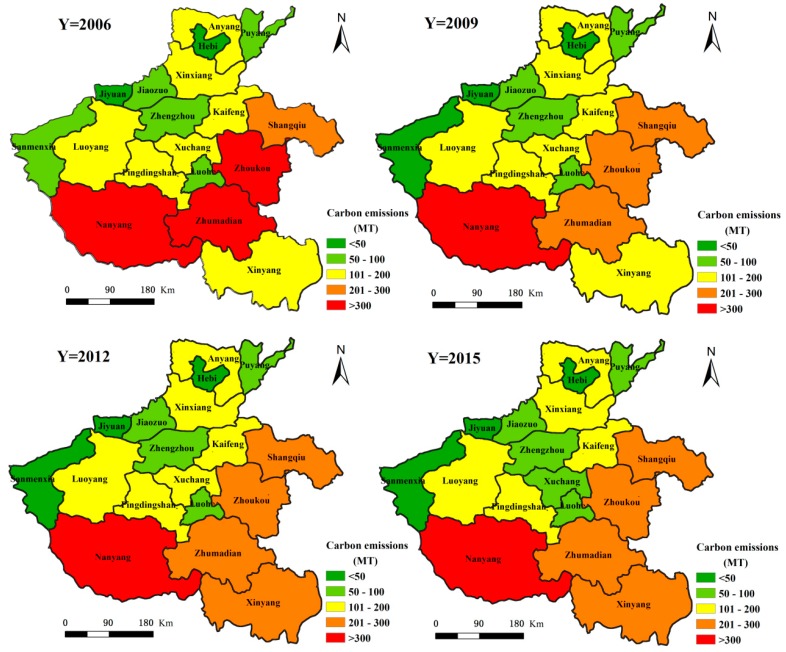
Spatial differences in carbon emissions from the primary sector in Henan Province during 2006–2015.

From 2006 to 2015, the spatial distribution pattern of carbon emissions in the primary sector had higher emissions in the southeast and lower emissions in the northwest (see Figure 4). Among them, the prefecture-level cities with higher carbon emission contributions are Nanyang City, Zhoukou City, Zhumadian City, Shangqiu City and Xinyang City, which respectively account for 13.29%, 11.31%, 10.68%, 10.03% and 8.18% of the total carbon emissions in the primary sector. Other prefecture-level cities with lesser carbon emissions are Jiyuan City, Hebi City and Sanmenxia City, which respectively account for 0.43%, 1.33% and 1.89% of the total carbon emissions in the primary sector. Nanyang City, which is known as the ‘Zhongzhou granary’ and the other four cities in Huang-huai (Zhoukou City, Zhumadian City, Shangqiu City and Xinyang City) are in the main crop production region. They combine together play a decisive role in the agricultural development of Henan Province. The crop yield from these five cities accounts for more than 50.00% of total crop production in Henan Province. Their animal farms account for about 50.00% of the total livestock product output in Henan Province. Concurrent to the high yield of agricultural crops, large amounts of fertilizer, pesticide, machinery and other agricultural production inputs are required. Additionally, high carbon emissions brought about by the respiration and excrement of large livestock numbers, make these five agricultural cities major contributors to primary sector carbon emissions. During 2006–2015, except for Xinyang City and Hebi City, where the primary sector carbon emissions showed an overall rising trend, the primary sector carbon emissions in the other prefecture-level cities showed an overall declining trend. Among them, the primary sector carbon emissions in Xinyang City increased from 1.96 million tons in 2006 to 2.03 million tons in 2015, corresponding to an increase of 3.64%. In Hebi City, the primary sector carbon emissions increased from 328,400 tons in 2006 to 334,500 tons in 2015, equal to an increase of 1.85%. The city with most significant reduction in primary sector carbon emissions was Nanyang City, with a 33.96% reduction from 4.61 million tons in 2006, to 3.04 million tons in 2015. The prefecture-level city with the next significant reduction in primary sector carbon emissions is Pingdingshan City, where the primary sector carbon emissions reduced from 1.56 million tons in 2006, to 1.06 million tons in 2015 causing a decrease of 32.24%. The main reasons leading to the differences in carbon emissions from the primary sector in these prefecture-level cities, is the increase in the irrigated area and crop acreage of Xinyang City and a smaller increase in the irrigated area of Nanyang City due to a significantly decreased crop seeded acreage.

#### 3.2.2. Spatial Distribution Characteristics of Carbon Emissions in the Secondary Sector in Henan Province

**Figure 5:**
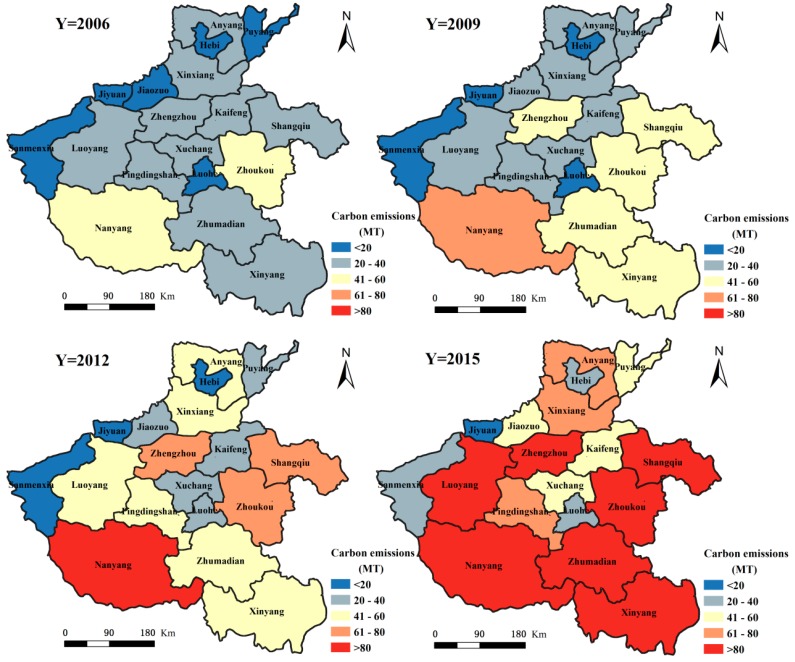
Spatial differences in carbon emissions from the secondary sector in Henan Province during 2006–2015.

Carbon emissions in the secondary sector in Henan Province between 2006 and 2015 showed varied spatial patterns (Figure 5). There were higher emissions in the northwest and lower in the southeast region. Here, the prefecture-level cities with higher secondary sector carbon emissions were Pingdingshan City, Luoyang City, Zhengzhou City and Anyang City, which respectively accounted for 18.86%, 11.50%, 9.94% and 9.85% of the total secondary sector carbon emissions in Henan Province, respectively. This distribution is closely related to the layout of the industrial development. The region along the Longhai and northern Henan is the core area of industrial development in Henan Province. The aforementioned cities are the main cities in this region. Their secondary sector energy consumption accounts for 40.00% to 50.00% of the total secondary sector energy consumption in the province. This large energy consumption leads to higher carbon emissions. During 2006–2015, apart from Jiaozuo City, Zhoukou City, Xuchang City and Anyang City, the carbon emissions in the secondary sector in the other cities increased gradually. The prefecture-level cities with a higher percentage increase in the carbon emissions from the secondary sector are Sanmenxia City, Kaifeng City, Pingdingshan City and Zhumadian City, where the emissions increased from 5.57 million tons, 1.93 million tons, 18.75 million tons and 1.94 million tons in 2006 to 10.87 million tons, 3.56 million tons, 33.63 million tons and 3.47 million tons in 2015, respectively. This corresponds to an increase of 95.26%, 84.25%, 79.34% and 79.09%, respectively. The prefecture-level city with the largest reduction percentage in secondary sector carbon emissions is Zhoukou City, where the emissions reduced from 1.72 million tons in 2006 to 553,400 tons in 2015, accounting for a decrease of 67.83%. The city with the second largest percentage reduction is Jiaozuo City, where the secondary sector carbon emissions reduced from 11.70 million tons in 2006 to 9.13 million tons in 2015. This reduction is mainly due to the rise in secondary sector production value in Zhoukou City and Jiaozuo City between 2006 and 2015, however as the secondary sector energy consumption is seen to decrease with year, it indicates that the energy efficiency of the secondary sector in Zhoukou City and Jiaozuo City has continued to increase.

#### 3.2.3. Spatial Distribution Characteristics of Carbon Emissions in the Tertiary Sector in Henan Province

**Figure 6:**
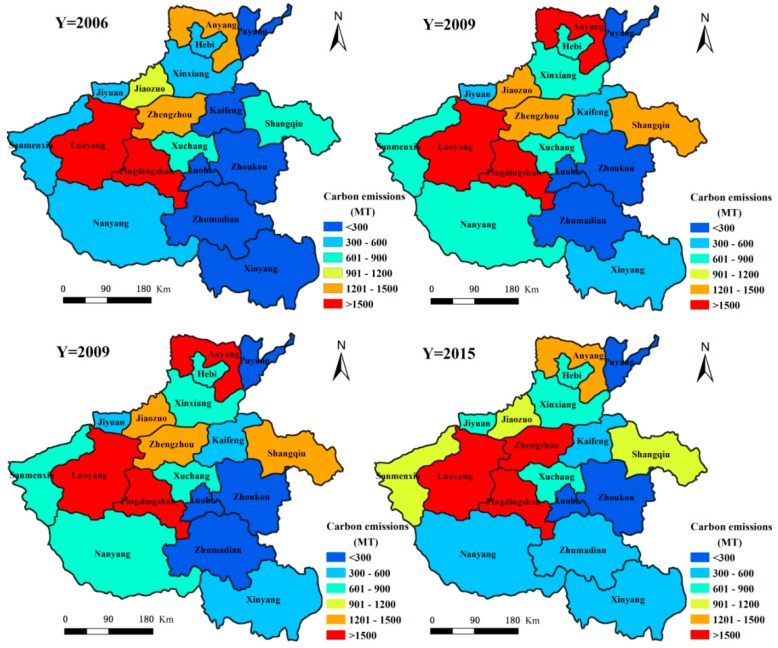
Spatial differences in carbon emissions from the tertiary sector in Henan Province during 2006-2015.

The spatial distribution of carbon emissions in the tertiary sector in Henan Province is shown in Figure 6. Between 2006 and 2015, the carbon emissions from this sector show an overall pattern of being higher in the southeast and lower in the northwest. Among the prefecture-level cities, those with the largest carbon emissions from the tertiary sector are Nanyang City, Zhoukou City and Zhengzhou City, whose emissions account for 10.69%, 9.86% and 8.94% of the total tertiary sector carbon emissions, respectively. This is due to the relatively higher energy consumption by the tertiary sector in these cities. Nanyang City is a historical, cultural and regionally central city in the Yu Shan-E area, its tourism and transportation are highly developed. It assumes that the tourism and transportation have contributed to the increase of carbon emission in its tertiary sector. Zhengzhou City, as the capital of Henan Province, is located in China’s geographical center. Its tertiary sectors, such as financial and service economy, are highly developed. They could be contributing to the high carbon emissions in the city. Zhoukou City is an important production base for grain, cotton, oil, meat and tobacco and has developed an active large-scale wholesale agricultural products market, which is dependent on the primary sector. Its carbon emissions have also increased accordingly. From 2006 to 2015, the carbon emissions from the tertiary sector in the various prefecture-level cities of Henan Province have increased annually. Of these, those with a higher increase in carbon emissions from the tertiary sector were Zhengzhou City, Hebi City and Jiyuan City, with increases from 340,700 tons, 67,800 tons and 31,600 tons in 2006 to 1.1 million tons, 207,600 tons and 94,300 tons in 2015, respectively. These correspond to a 263.27%, 206.55% and 198.00% increase for the three cities respectively. The prefecture-level cities with a relatively small increase in the carbon emissions from the tertiary sector are Zhoukou City and Zhumadian City. Here, the tertiary sector carbon emissions increased from 467,400 tons and 365,300 tons in 2006 to 1,139,200 tons and 899,500 tons in 2015, amounting to an increase in 143.77% and 146.26%, respectively. The highest increase here is about twice the minimum increase.

### 3.3. Evaluation of Economic Development Coordination Degree in Henan Province

The coordination coefficient of industrial development from the perspective of carbon emissions in Henan Province in 2006–2010 increase by 1.34%, while it decreases by 8.02% in 2011–2015. The imbalance of industrial development is the main reason for this phenomenon. In 2006–2015, the industrial development gap is gradually expanded in Henan Province. In 2011–2015, industrial GDP growth rate is extremely poor, expanding by 22.13%. From the perspective of carbon emissions, the carbon emissions of the secondary industry in Henan Province shows a downward trend in 2011–2015, which lead to a slowdown in the GDP growth rate of the secondary industry and is 79.10% lower than the 2006–2010 GDP growth rate. The increase in carbon emission growth rate increases the GDP growth rate of the tertiary industry, that is, the change in the carbon emission structure lead to changes in the industrial structure, resulting in a prominent polarization of industrial development, which in turn reduce the overall coordination of industrial development in Henan Province.

**Figure 7:**
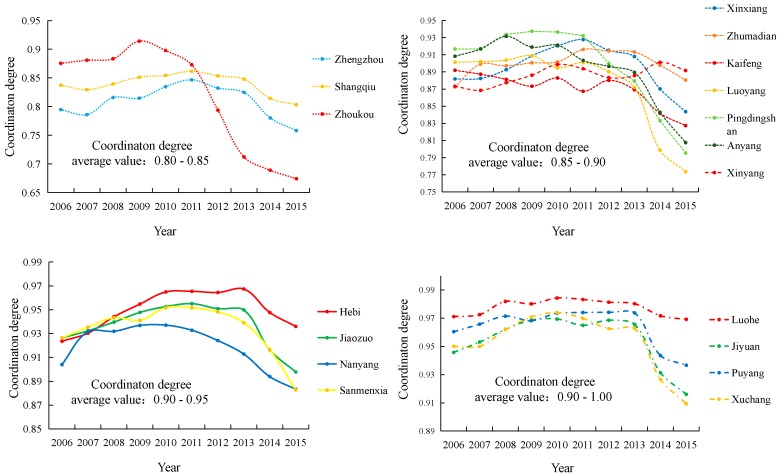
Evaluation of economic development coordination degrees in Henan Province from 2006 to 2015.

On this basis, the economic development coordination degree of various cities in Henan Province is calculated (Figure 7). Zhengzhou City has the lowest degree of coordination of economic development, with the coordination degree reduced from 0.79 in 2006 to 0.75 in 2015, the carbon emissions generated by unit economic growth in Zhengzhou City are significantly higher than the provincial average. In 2015, the carbon emissions generated by unit economic growth in Zhengzhou City was 2.19 times that of Henan Province. In 2006–2011, the GDP growth rate of the secondary industry in Zhengzhou City reaches 168%, which is higher than the GDP growth rate of the tertiary industry by 40%, the carbon emission growth rate is 34%, which is lower than the growth rate of the tertiary industry’s carbon emission by 71%. However, the GDP growth rate of the tertiary industry in 2012–2015 is higher than that of the secondary industry by 41%. The change in carbon emission structure causes the industrial structure of Zhengzhou to change, which led to the break of the balanced development of the industry and the widening of the industrial development gap and led to a gradual decline in the coordination of industrial development in Zhengzhou.

The main reason is that it has seen a rapid economic growth in the Central Plains Economic Zone. The per capita GDP has seen an increase from 34,069 yuan/person in 2006, to 72,992 yuan/person in 2015. Simultaneously, the total carbon emissions in Zhengzhou City have increased from 14.18 million tons in 2006 to 20.10 million tons in 2015. It can be concluded that as the per capita GDP doubled, the carbon emissions grew by half. This rapid economic development is however at the cost of the environment. The decline rate of the coordination degree in Zhoukou City is the most rapid. The coordination degree of development decreased by 22.99% from 0.87 in 2006 to 0.67 in 2015. In the process of industrial economic development in Zhoukou, the industrial carbon emission structure changes too fast and the carbon emission structure does not match the industrial economic structure. The carbon emission growth structure changes from the primary industry to the tertiary industry. In 2010–2015, the industrial GDP growth rate of Zhoukou City increases by 81% compared with 2006–2009. The change in carbon emission structure increases the gap between industries. A marked decline in the coordination degree of the economic development between the various prefecture-level cities in Henan province is seen. Here, not only are the coordination degrees of development quite different but the differences in the differences in variability are also large.

## 4. Policy Implications

As a large agricultural province, Henan Province has a large population and a relatively fast GDP growth. In recent years, with the gradual convenience of transportation, food production has promoted the rapid development of social economy in Henan Province, which has made Henan a hot spot for industrial economic activities. The three major economic sectors in Henan province will continue to maintain a steady growth in the foreseeable future. While facing the continuous growth of the population, Henan Province must strive to maintain its status as a major agricultural province. In order to achieve a coordinated economic and environmental development, based on the above conclusions, the study presents the following recommendations to promote the realization of low-carbon economic development goals in Henan Province:

(1) The “13th Five-Year” plan in China clearly stated necessary responses to global climate change. It places equal emphasis on mitigation and adaptation of carbon emissions, emission reduction commitments, the ability to adapt to climate change, the depth of participation in global climate governance and the contributions to addressing global climate change. Henan province is a province with a large population. It is also a big economic and agricultural entity in the nation. In order to curb carbon emissions in Henan, focus should be paid on the effective control of industrial growth and usage of electric power, building materials (iron/steel), chemicals and other sources of carbon emissions. In the meantime, promotion of low carbon industry, energy, construction, transportation are other key strategies. From an economic structure perspective, Henan Province should further optimize the economic structure. The economic structure could transform from its over-reliance on the secondary sector to an environmentally friendly, green-driven and low-carbon sector. For each prefecture-level city, energy-saving emission reduction work should be deployed by referencing to the actual situation of economic development in the city and the local conditions.

(2) Economic development from scientific and technological progress is the internal driving force to promote a sustainable regional development. Considering the relationship between carbon emissions and industrial economic development, Henan Province should also pay attention to the rational transformation of carbon emission structure while transforming industrial structure, that is, it should consider adapting technological innovation and energy structure adjustment to optimize the transformation of industrial structure. It should be carried out simultaneously to achieve emission reduction targets. The development of industry should be carried out simultaneously. The development of high-coordination industry can improve the overall economic level of the region. Adjusting the use of energy structure is an effective way to reduce carbon emissions in human activities. Only industrial structure and carbon emission structure are carried out simultaneously. Transformation can promote the future sustainable development of the region. 

(3) Institutional guarantee is one of the necessary ways to reduce carbon emissions. Henan Province should carry on different possible low carbon pilot projects and implement the demonstration projects of near zero carbon emission. We should actively promote the trading market of carbon emission, carbon emissions reporting system and the verification, certification and quota management of key emission units. Henan should improve the system of statistical accounting, evaluation and accountability and improve the standard system of carbon emission. Other regions in the world with similar population and economic issues could learn the experience of emission control in Henan and actively participate in the negotiation of global climate change and promote the establishment of a fair, reasonable and win–win global climate governance system.

## 5. Conclusions

Based on the differentiated calculation for primary, secondary and tertiary sectors. This study has analyzed the temporal and spatial changes to carbon emissions in the three major economic sectors in Henan Province from 2006 to 2015. Further, a sustainable development evaluation was performed to explore the relationship between the economic development and environmental protection. The interpretations can be summarized as follows:

(1) The growth rate of carbon emissions in China and Henan province is 1.28 times and 1.08 times of the world average carbon emissions respectively and the proportion of the third industry is 19.93% and 29.92% lower than the world average, differences in industrial structure have an important impact on the growth of regional carbon emissions. The temporal changes in carbon emissions in the three major economic sectors in Henan Province highlighted that the total carbon emissions and the carbon emissions alone in the secondary sector initially increased and then decreased. The carbon emissions from the tertiary sector showed a continued increasing trend. As opposed to those in the secondary sector, the primary sector declined gradually. As the production value of the secondary industry in Henan Province contributed towards 62.00% of the province’s GDP, the contribution of the carbon emissions in the secondary sector towards the total carbon emissions from the three major economic sectors in the province was 82.00%. Consequently, the total carbon emissions in Henan Province showed the same variation trend as the secondary sector. Since the contribution of the tertiary sector production value in Henan Province increased from 27.00% in 2006 to 32.00% in 2015, its carbon emissions also increased due to the sector development. The carbon sequestration in the primary sector declined, as the farmland ecosystem played an important carbon sink function in the carbon emissions of Henan Province.

(2) The spatial distribution of carbon emissions in the primary and tertiary sectors showed higher values in the southeast and lower values in the northwest regions from 2006 to 2015. The carbon emissions in the secondary sector revealed higher values in the northwest and lower values in the southeast overall. The spatial disparities in cities at different prefecture-level do not only lie on the quantity of carbon emissions from the three sectors of economy but also the large variation with regards to the change in quantity of carbon emissions. Carbon emissions in the primary sector are more concentrated in the southeast region as this region is the central area for agricultural development. At the same time, based on the highly developed agriculture in the southeastern region, the formation of a large wholesale market for agricultural products has resulted in the increase of carbon emission in the tertiary sector. The spatial distribution of carbon emissions from the secondary sector is closely related to the layout of the industrial development in Henan Province. The areas along the Longhai and Northern Henan are the core regions of industrial development causing relatively higher carbon emissions from the secondary sector in these cities.

(3) From 2006 to 2015, the coordination degree of economic development in various prefecture-level cities of Henan Province showed a decrease, although the coordination of economic and environmental development in most areas was good. The differences in the coordination degree of development and variation amplitude for various prefecture-level cities is large. Among them, Zhengzhou City showed the lowest coordination degree of economic development and Zhoukou City showed the highest reduction rate of coordination degree. The results indicate that the more similar the carbon emission structure is to the industrial structure, the higher the coordination of industrial development, that is, the mismatch between the carbon emission structure and the industrial structure transformation in the process of industrial development is the main reason for the reduction of regional industrial coordination.

## Figures and Tables

**Table 1:** Indirect carbon emission index for agricultural capital investment.

Types	Unit	Coefficient
Nitrogen fertilizer	kg C/kg N	2.12
Phosphatic fertilizer	kg C/kg P_2_O_5_	0.64
Potash fertilizer	kg C/kg K_2_O	0.18
Pesticide	kg C/kg	6.00
Agricultural film	t CO_2_/t	2.58
Agricultural irrigation	kg C/hm^2^	25.00
Agricultural cultivation	kg C/hm^2^	31.06

**Table 2:** Straw combustion coefficients.

Types	Coefficient of Straw	Collection Coefficient of Straw	Carbon Emission of Straw kg C/kg
Cereal	1.0	0.89	0.09
Wheat	1.1	0.89	0.06
Corn	2.0	0.91	0.04
Other cereals	1.6	0.90	0.07
Soybean	1.6	0.95	0.06
Other beans	1.6	0.90	0.07
Sweet potato	0.5	0.95	0.02
Peanut	1.5	0.95	0.04
Rapeseed	3.0	0.90	0.068
Sesame	3.0	0.90	0.068
Cotton	1.69	0.91	0.112
Tobacco	0.71	0.95	0.032
Mushroom	-	-	-

**Table 3:** Emission coefficients for CH_4_ and N_2_O emissions from livestock.

Types	(kg/head/year) CH_4_ Emission from Animal Digestive Tract	(kg/head/year) CH_4_ Emission from Animal Excretion	(kg/head/year) N_2_O Emission from Animal Excretion
Dairy cattle	89.70	8.33	2.07
Cattle	67.90	3.31	0.85
Horse	18.00	1.64	0.33
Donkey	10.00	0.90	0.19
Mule	10.00	0.90	0.19
Pig	1.00	5.08	0.18
Goat	9.40	0.28	0.11
Sheep	8.70	0.26	0.11
Poultry	0.00	0.02	0.01
Rabbit	0.25	0.08	0.02

**Table 4:** Energy consumption carbon emission conversion factor.

Fuel Type	Emission Factor per MJ	Conversion Factor	Oxidation Rate	Lower Heating Value
Crude coal	25.80	0.71 tce/t	0.92%	20.91
Coke	29.20	0.97 tce/t	0.93%	28.44
Crude oil	20.00	1.43 tce/t	0.98%	41.82
Diesel	20.20	1.46 tce/t	0.98%	42.65
Kerosene	19.60	1.47 tce/t	0.98%	43.07
Gasoline	18.90	1.47 tce/t	0.99%	43.07
Fuel oil	21.10	1.43 tce/t	0.99%	41.82
Natural gas	15.30	1.33 tce/10^3^ m^3^	0.99%	38.93

**Table 5:** CO_2_ emission factor from IPCC and this study.

Fuel Type	CO_2_ Emission Factor (kgCO_2_/kg or kgCO_2_/m^3^)	IPCC CO_2_ Emission Factor (kgCO_2_/kg or kgCO_2_/m^3^)
Crude coal	1.82	2.69
Coke	2.83	2.69
Crude oil	3.01	2.76
Diesel	3.08	2.73
Kerosene	3.03	2.56
Gasoline	2.95	2.26
Fuel oil	3.20	2.98
Natural gas	2.16	2.33
